# Diving ergospirometry with suspended weights: breathing- and fin-swimming style matter

**DOI:** 10.1007/s00421-022-05009-y

**Published:** 2022-08-25

**Authors:** Andreas Koch, Dennis Kramkowski, Mattes Holzum, Wataru Kähler, Sebastian Klapa, Bente Rieger, Burkhard Weisser, Jochen D. Schipke

**Affiliations:** 1 German Naval Medical Institute, Kiel, Germany; 2 Dept. of Sportsmedicine, Christian-Albrechts-University, Kiel, Germany; 3 Institute of Physiology, Christian-Albrechts-University, Kiel, Germany; 4 Institute of Experimental Medicine, Christian-Albrechts-University Kiel, Kronshagen, Germany; 5 Research Group Experimental Surgery, University Hospital Düsseldorf, Düsseldorf, Germany

**Keywords:** Diving, Efficiency, Bottom time, Fitness, Hyperbaric chamber

## Abstract

**Purpose:**

Scuba diving is a complex condition including elevated ambient pressure, limited air supply, increased breathing work, and unfamiliar fin-swimming. Earlier approaches to assess diving specific data did not comprehensively address these aspects. We first present an underwater ergospirometry system and then test the hypothesis that both breathing characteristics and fin-swimming style affect the air consumption.

**Methods/Participants:**

A suspended-weights ergospirometry system was mounted inside a hyperbaric chamber. Ergo group: 25 divers (24.6 ± 4.1 years); three set-ups: dry normobaric cycling (75–225 W), dry cycling at 20 m simulated depth (75–225 W), fin-swimming at 20 m (5–8 kg suspended weights). Style group: 20 other divers (24.6 ± 4.1 years): fin-swimming at 20 m (5–8 kg) with regard to ventilation ($\dot{V}$E) and fin-swimming style.

**Results:**

Ergo group: linear heart rate and oxygen uptake ($\dot{V}$O_2_) increases with both 50 W-bicycle steps and suspended-weights ergometry (*r* = 0.97). During hyperbaric conditions, $\dot{V}$E was less increased versus normobaric conditions. Style group: the more efficient hip/thigh-oriented style shifted towards the knee/calf-oriented style. $\dot{V}$E and $\dot{V}$O_2_ were higher in beginners (< 100 dives) versus advanced divers (≥ 100 dives). Significant differences on the 5 kg-step: $\dot{V}$E: 31.5 ± 7.1 l/min vs. 23.7 ± 5.9 l/min and $\dot{V}$O_2_: 1.6 ± 0.3 l/min vs. 1.2 ± 0.3 l/min. A comparison is presented, in addition to illustrate the impact of differences in breathing characteristics and fin-swimming style.

**Conclusions:**

Diving ergospirometry with suspended weights in a hyperbaric chamber allows for comprehensive studies. Little diving experience in terms of breathing characteristics and fin-swimming style significantly increases $\dot{V}$E thereby increasing the risk of running-out-of-air.

## Introduction

One of the most dangerous risks of scuba diving is suddenly running-out-of-air. Such a dangerous situation might happen due to an unexpectedly high ventilation during fin-swimming at depth, possibly resulting in an emergency ascent and severe diving accident (Denoble et al. 2008).

Fin-swimming is a well-established sports discipline, and fin-swimming during scuba diving under different conditions has already been extensively investigated (Pendergast et al. 1996) (Pendergast et al. 2005). However, sports physiological measures as ventilation ($\dot{V}$E) and oxygen uptake ($\dot{V}$O_2_) of fin-swimming while scuba diving in hyperbaric conditions are still unknown. Ergometric test devices for cycling, running as well as rowing are available worldwide as commercial off-the-shelf systems, but a device addressing the complex situation of professional and sports diving does not exist until now. Yet, a number of partial solutions has already been presented (Thalmann et al. 1978, 1979), like submersed bicycle ergometry with $\dot{V}$O_2_-measurements (Almeling et al. 2004, 2006; Vinetti et al. 2021). Ergospirometric measurements during fin-swimming with scuba have also been performed before (Simon et al. 1983; Niklas and Peter 1993; Bräuer et al. 1994; Pendergast et al. 1996, 2005), but they took place in pools or swim-channels at shallow depths or while head-out (Shykoff 2009) or fully submersed at greater depths (Held and Pendergast 2013) underwater bicycling. The additional effects on pulmonary function and increased breathing work at depth (Maio and Farhi 1967; Wright et al. 1972) were addressed in only few studies (Ray et al. 2008).

**Fig. 1:**
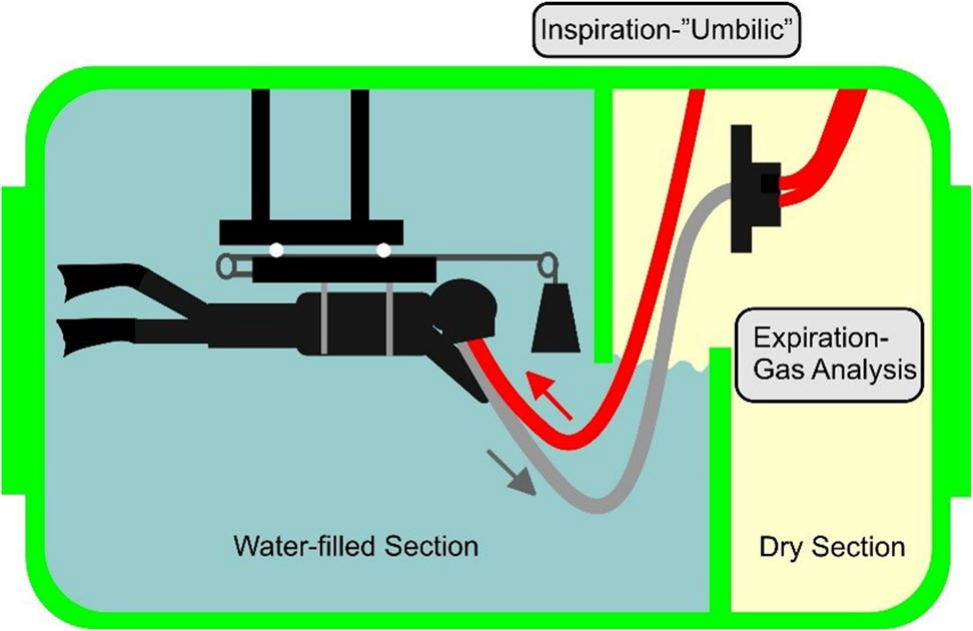
Schematic of the HYDRA hyperbaric chamber that, due to a Buffalo wall (Lanphier and Morin 1970), contains a wet and a dry section. For performing diving ergospirometry, a suspended-weights system was used from both the first group (ergo group) and the second group (style group)

At the German Naval Medical Institute, a completely redesigned diving-ergospirometry system using suspended weights was installed inside a hyperbaric chamber with a Lanphier–Morin barrier (= Buffalo-wall; Lanphier and Morin 1970) (see Fig. 1). This system permits fin-swimming at varying loads up to simulated depths of 90 m (≙ 10 bar ambient pressure).

In this study, details of that system are presented together with sports physiological aspects of fin-swimming.

Some details of this novel ergometer system shall be explained. The suspended-weights system allows for stationary fin-swimming. This latter technique is an accepted method in elite swimmers´ training for measuring $\dot{V}$O_2_ during swimming in parallel to conventional swim tests (O’Toole and Douglas 1995; Ueda and Kurokawa 1995; Hooper et al. 1998). Both in the tethered swimming- and the suspended-weights ergometry, the effective workload is not defined by the velocity over ground but by the weight force of the counterbalancing suspended weights. This mirrors the effective amount of locomotor force that must be generated by the swimmer or diver.

To our knowledge, a computerised system with suspended weights has not been used before in a wet hyperbaric chamber, it was our first aim to compare this diving ergospirometry, i.e. fin-swimming at 3 bar, with regular dry bicycle ergospirometry both at 1 bar and at 3 bar ambient pressure in one group of young and experienced divers. Measurement of physiological parameters, i.e. heart rate (HR), $\dot{V}$E, $\dot{V}$O_2_, carbon-dioxide production ($\dot{V}$CO_2_), blood gases, pH, lactate and an index of efficiency (Ix_Eff_), should also allow for comparison with other disciplines.

It was the second aim of the study on another group of young and experienced divers to assess fin-swimming style dependent changes in $\dot{V}$E, $\dot{V}$O_2_, and Ix_Eff_ during incremental exercise at a simulated depth of 20 m. Thus, this part was intended to provide both information on who might be at risk of unexpectedly running-out-of-air and test our hypothesis that breathing characteristics and fin-swimming style would impact the breathing gas consumption.

## Participants and methods

### Participants

The ergo group consisted of 25 healthy, male, experienced military divers (24.6 ± 4.1 years; 181.6 ± 6.9 cm; 77.5 ± 8.7 kg; BMI: 23.6 ± 1.6 kg/m^2^; > 100 dives) (mean ± SD). The style group consisted of 20 healthy, male, military divers (25.5 ± 3.5 years; 183.6 ± 8.1 cm; 79.9 ± 10.5 kg; BMI: 23.6 ± 1.9 kg/m^2^). These participants had a wider range of diving experience (16 to 831 dives). Two subgroups were formed and were termed ‘beginners’ (*n* = 13; < 100 dives) and ‘advanced’ divers (*n* = 7; ≥ 100 dives). All participants were examined by a diving doctor for fitness-to-dive and had given written informed consent. The study was approved by the Ethics Committee of the Christian-Albrechts-University, Kiel, Germany.

### Diving ergospirometry

The study took place in the hyperbaric chamber facilities (HYDRA 2000) of the German Naval Medical Institute, Kiel-Kronshagen. A so-called “Buffalo wall” separates the water-filled section from the dry section inside the hyperbaric chamber (see Fig. 1). The chamber has a total length of 5.30 m and a diameter of 2.50 m. The wet section has a length of 4.30 m and thus, allows for fin-swimming under stationary conditions. A pneumotachograph and a Douglas bag were installed in the dry section of the chamber (Fig. 1).

Fin-swimming ergometry was carried out using a suspended-weights system, which was mounted on the ceiling of the chamber. The diver was fixed with belts to a pullback-mechanism that kept him in a horizontal swimming position. Pieces of lead with different weight forces were visible in front of the diver. To compensate the retraction force of the weights and to stay stationary, the diver needed to fin swim with different intensity.

The spirometric device consisted of a semi-open Douglas bag system (EosSprint, Jäger, Germany) and a full-face diving mask (Interspiro, Germany). A modification of the breathing regulator allowed for the collection of the exhaled gas via a hose to the pneumotachograph and the Douglas bag. Measurements of the exhaled gas samples (O_2_- and CO_2_-fraction) were performed with the EosSprint-system and were carried out through a pressure reducer outside the hyperbaric chamber (Fig. 1). For capillary blood gas analysis, the first sample was always taken at ambient normobaric pressure before the onset of the three protocols. The second sample was taken at the corresponding pressure immediately after completion of the task.

Heart rate was continuously assessed. To achieve steady states, each workload step lasted for 3 min. This was important during the hyperbaric exposure, because the 3-m-long expiration hose (*Ø*: 5 cm) caused about a 2-min delay for O_2_- and CO_2_ fractions to become stable. Thus, a mean value from within the third minute of each loading step is reported.

Dynamic valves in the expiration hose maintained a minimal positive pressure inside the diving mask (3–5 cmH_2_O). This kept the system dry during diving, which was mandatory for correctly measuring the ventilation. Unlimited air was supplied from directly outside the chamber.

### Test settings

*Ergo group* Each diver performed three ergospirometry runs on successive days in random order to avoid systematic test errors. Two test runs were performed on a regular bicycle-ergometer (Jäger-ER900, Jäger, Germany) inside the dry section of the hyperbaric chamber, one under normobaric conditions (1 bar), the other at a simulated depth of 20 m (≙ 3 bar).

This depth was chosen, because it is a very common diving depth in professional and recreational diving. Diving ergometry was also performed at a depth of 20 m. All divers used the same fin type to avoid fin type dependent effects on the index of efficiency (Ix_Eff_) (Pendergast et al. 2003). The same ergospirometric device including the full-face mask was used in all three set-up runs to ensure identical conditions for the analysis of the exhaled air. Additionally, blood samples were taken from the earlobe for blood gases and pH (50 µl; ABL500, Germany) and for lactate (10 µl; Dr. Lange Photometer, Berlin, Germany). In each case, the first sample was taken at rest, under normobaric dry and hyperbaric dry conditions (bicycle), as well as before fin-swimming at 20 m simulated depth, the second sample within the first minute after completion of exercise, respectively.

Consistent with regular fitness tests in young, healthy men with normal BMIs, the bicycle ergometry consisted of five steps (rest, 75–125–175–225 W).

Diving ergometry began with a retraction weight of 5 kg. This weight was chosen as the result of preceding preliminary tests with very experienced divers. These tests showed that 5 kg weight caused the same HR as a load of 75 W on the bicycle. Adding another kilogram happened to cause the same HR-increase as 125 W on the bicycle. Thus, each additional kilogram was equivalent with additional 50 W. In addition to this reliable relation, the divers were asked to describe the perceived level of exertion according to the Borg scaling (Borg 1982). Remarkably, the 5-kg was perceived as 75 W on the bicycle, and each additional kilogram was perceived as additional 50 W.

Thus, the retraction weight was increased by 1 kg every 3 min to a maximum of 8 kg. Directly after completion of the task, the diver left the water-filled part of the chamber, but continued breathing with the full-face mask to allow capillary blood-sampling early during recovery, i.e. within the first minute after the end of exercise. In total, 5 min were used to collect data during the recovery. Thereafter, decompression was started.

To avoid possible effects due to anxiety or expectation, all divers of the entire study were carefully informed in advance about changes in workloads.

*Style group* This group performed fin-swimming ergospirometry at 3 bar that was carried out in the same way as described before for both beginners and advanced divers.

For this group, an index of efficiency was calculated from the workload (= weights) during fin-swimming and from the oxygen uptake during each 3-min weight steps (see formula (1)).

1 $${\text{Index of efficiency}} = \left( {{\text{weight}} \times 1000} \right)/\left( {E{-}e} \right),$$

with ‘weight’ ranging from 5 to 8 kg, E: overall energy consumption [kJ] during exercise minus, e: energy consumption at rest [kJ] (both calculated from the oxygen uptake [l/min]. Values were used from the last 30 s out of the 3-min load step, where measurement of $\dot{V}$O_2_ from the gas samples out of the Douglas-bag was stable. The caloric equivalent for mid-European balance mixed diet was assumed to be 20.5 kJ/l O_2_.

Additionally, all in-water runs were recorded with a video system and stored on a computer for later analysis of the individual swimming style.

### Video analysis

**Fig. 2:**
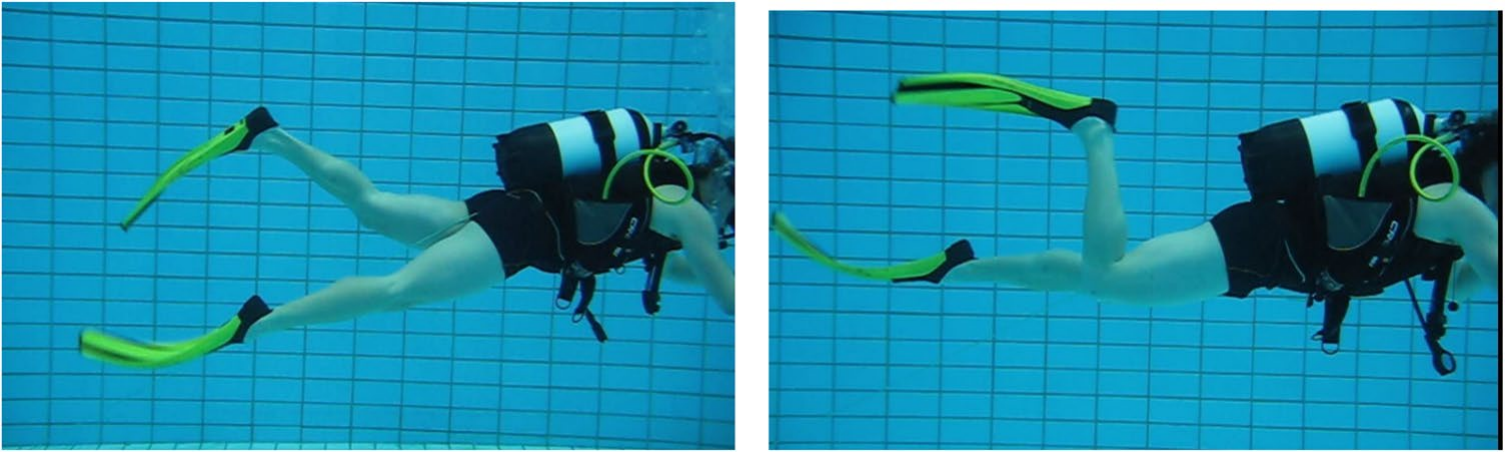
Fin swimming styles. Left: hip/thigh style; Right: knee/calf style

Two styles were distinguished: the hip/thigh-oriented style and the knee/calf-oriented style, the former being reported to be more efficient (Fig. 2).

The videos of the fin-swimming ergometry were examined by three independent investigators. During each 3-min workload step, the positions of hip joint, knee joint and ankle were rated twice (at 00:30 min and 02:30 min) as hip/thigh-oriented (3 points), mixed (2 points) or knee/calf-oriented (1 point). Then, both time points were averaged for each joint and investigators. The mean values from the three investigators were added up to values ranging from a maximum 9 points (all joint-positions typical for the hip/thigh-oriented style) to a minimum 3 points (completely knee/calf-oriented swimming style). In case workload steps were not completed due to exhaustion, they were rated as zero. As some divers could not complete all workload steps, some final ratings fell below the 3-point minimum for the fin-swimming style.

### Comparison of calculated bottom times

To demonstrate the effects of diving experience on a calculated bottom time, a comparison is provided between the individual results ($\dot{V}$E, $\dot{V}$O_2_, and Ix_Eff_) of the most (831 dives) and the least (16 dives) experienced diver. An air supply of 2800 l (= 27 l; 200 bar) was assumed. As the dive took place on 20 m, $\dot{V}$Es were multiplied by a factor of ‘3’.

### Statistics

Using standard software (SPSS Statistics 25, IBM, New York, U.S.) two-way ANOVAs with repeated measures on the factors condition (1-bar bike; 3-bar bike; 3-bar fin) and load (rest, 75–125–175–225 W) were calculated. Single variables were tested using the t-test for dependent samples. Where necessary, Bonferroni-correction was employed. Differences were considered significant with *p* < 0.05.

## Results

### Ergo group

**Fig. 3:**
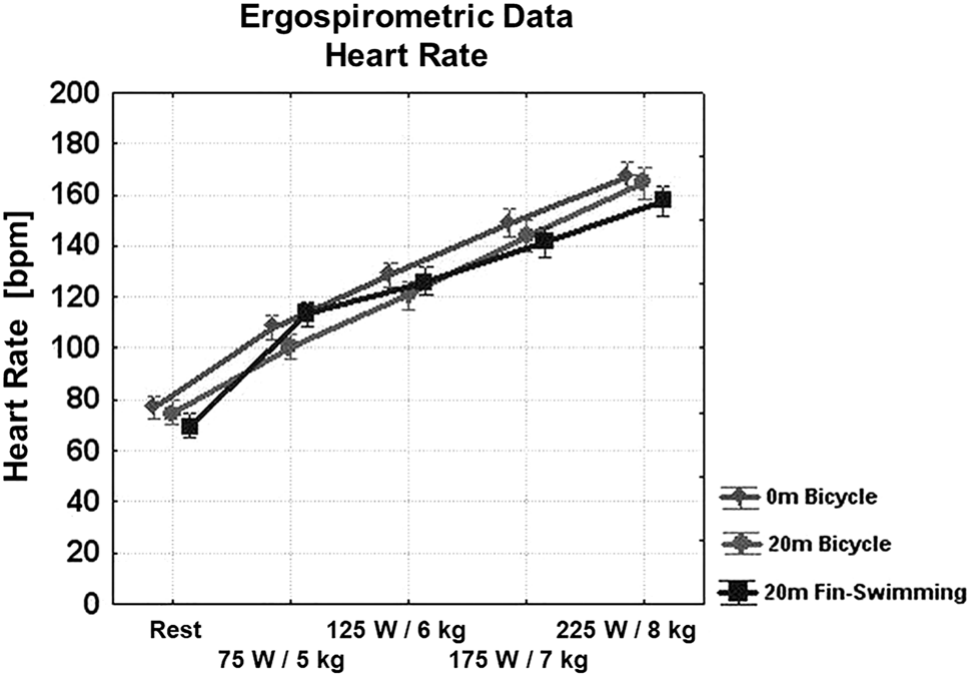
Ergo group: HR during the three set-ups, i.e. 1-bar and 3-bar bicycle ergometry and 3-bar fin-swimming ergometry. Nearly linear relationship between workload and HR in all three set-ups, no significant differences between the conditions. (Mean values ± SD)

**Fig. 4:**
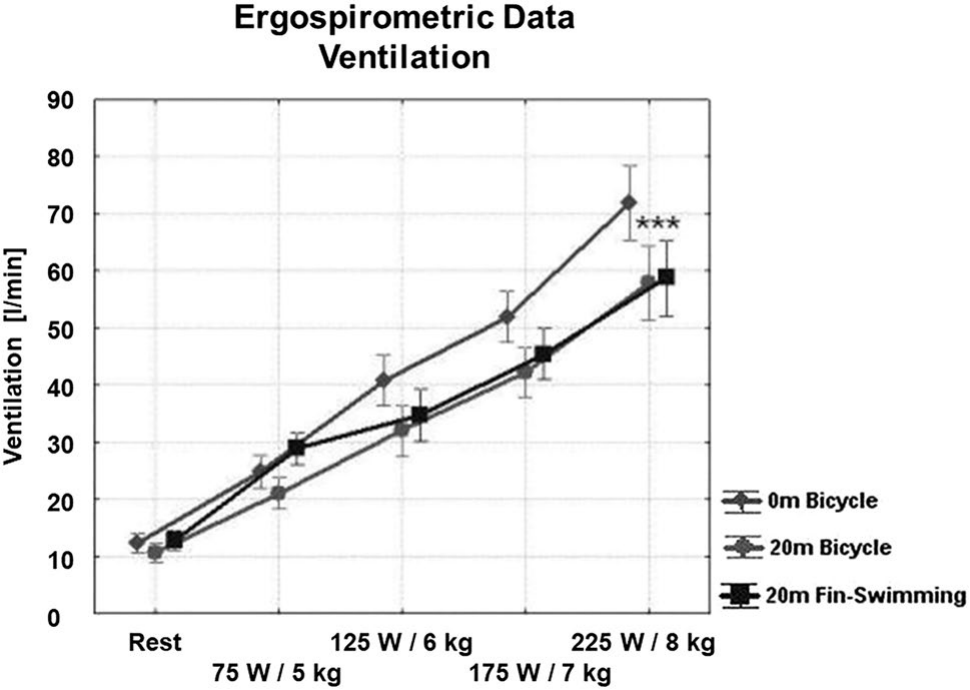
Ergo group: ventilation during the three set-ups. Significantly lower ventilation on both 3-bar conditions at the highest workload compared with 1-bar cycling (*p* < 0.05). (Mean values ± SD)

**Fig. 5:**
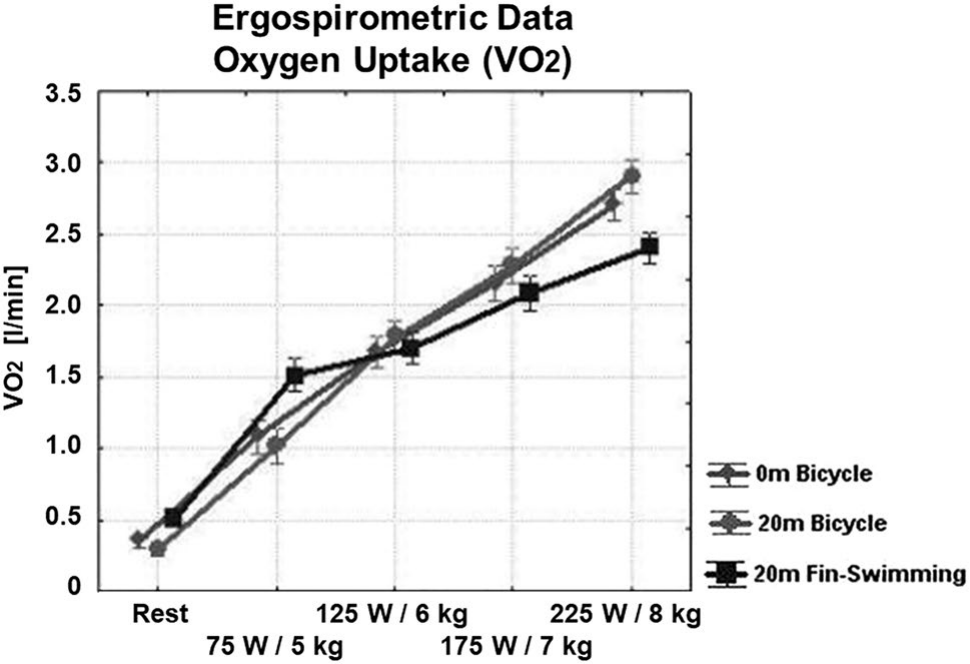
Ergo group: $\dot{V}$O_2_ during the three set-ups. Nearly linear relationship between workload and $\dot{V}$O_2_ in all three set-ups; no significant differences between the conditions. (Mean values ± SD)

**Fig. 6:**
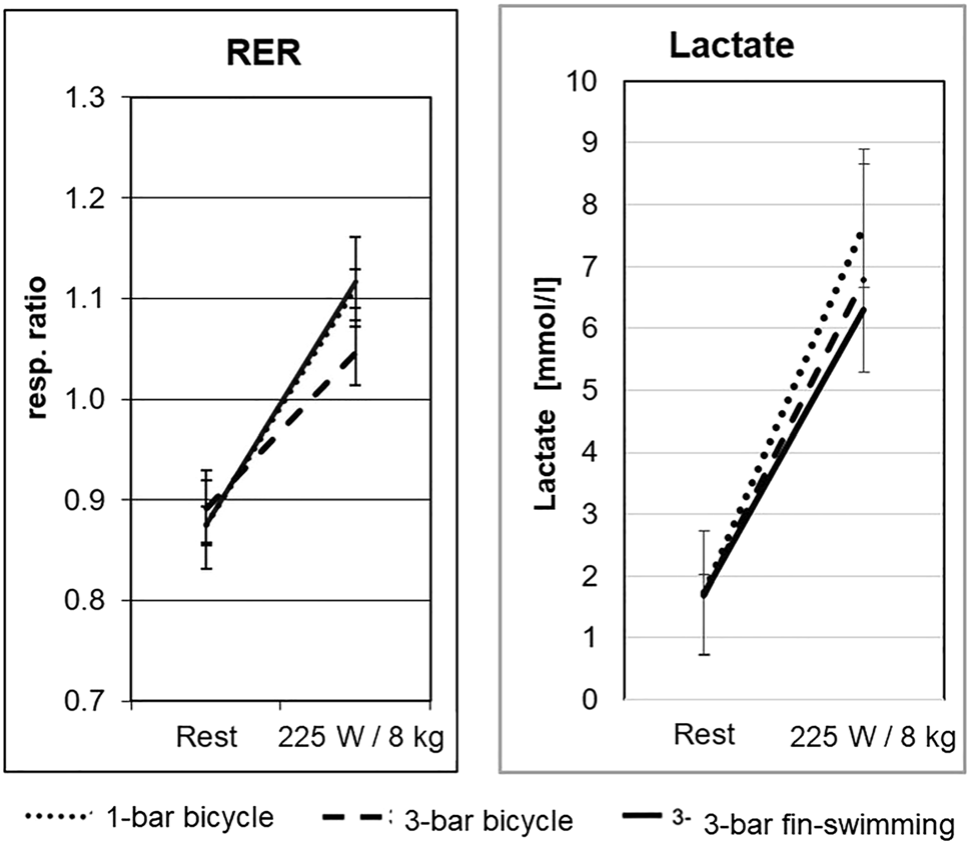
Left: Ergo group: respiratory exchange ratio (RER) at rest was in a normal range for all three set-ups. At the highest load RER had significantly increased for all three set-ups, suggesting an increased $\dot{V}$CO_2_ at high load (*p* < 0.05). (Mean values ± SD). Right: lactate levels for all three set-ups at rest were within the normal range. At the highest load, they were all significantly increased. The increase for the 1-bar bicycle ergospirometry was significantly higher over both 3-bar conditions (*p* < 0.05). (Mean values ± SD)

During 1-bar cycling and at the highest load, HR reached 167 ± 3/min (mean ± SD) (Fig. 3), $\dot{V}$E 72 ± 13 l/min (Fig. 4), $\dot{V}$O_2_ 2.7 ± 0.1 l/min (Fig. 5), $\dot{V}$CO_2_ 3.0 ± 0.1 l/min (not shown), the latter two variables giving a RER of 1.11 ± 0.02 (Fig. 6, left). Blood lactate was 7.66 ± 0.55 mmol/l (Fig. 6, right) after the highest load, which was suggestive of anaerobic conditions.

Cycling at 3 bar was comparable with normobaric cycling at 1 bar with a peak HR of 163 ± 4/min, $\dot{V}$E 58 ± 22 l/min, $\dot{V}$O_2_ 2.9 ± 0.1 l/min, $\dot{V}$CO_2_ 3.0 ± 0.1 l/min, and RER 1.05 ± 0.03. Blood lactate after the highest load was 6.77 ± 0.55 mmol/l, and thus slightly lower compared to 1-bar cycling (see Figs. 3, 4, 5, and 6).

For 3-bar fin-swimming, the results were comparable to cycling at either pressure: HR reached 158 ± 3/min, $\dot{V}$O_2_ 2.4 ± 0.1 l/min, $\dot{V}$E 59 ± 9 l/min, $\dot{V}$CO_2_ 2.7 ± 0.1 l/min, and RER 1.12 ± 0.04. No significant differences were found between the three set-ups, indicating that the conditions were comparable in their physical strain. However, $\dot{V}$E during 175 and 225 W was significantly lower during both hyperbaric conditions compared to normobaric cycling with no differences between cycling and fin-swimming at 3 bar (see Figs. 3, 4, 5, and 6).

**Fig. 7:**
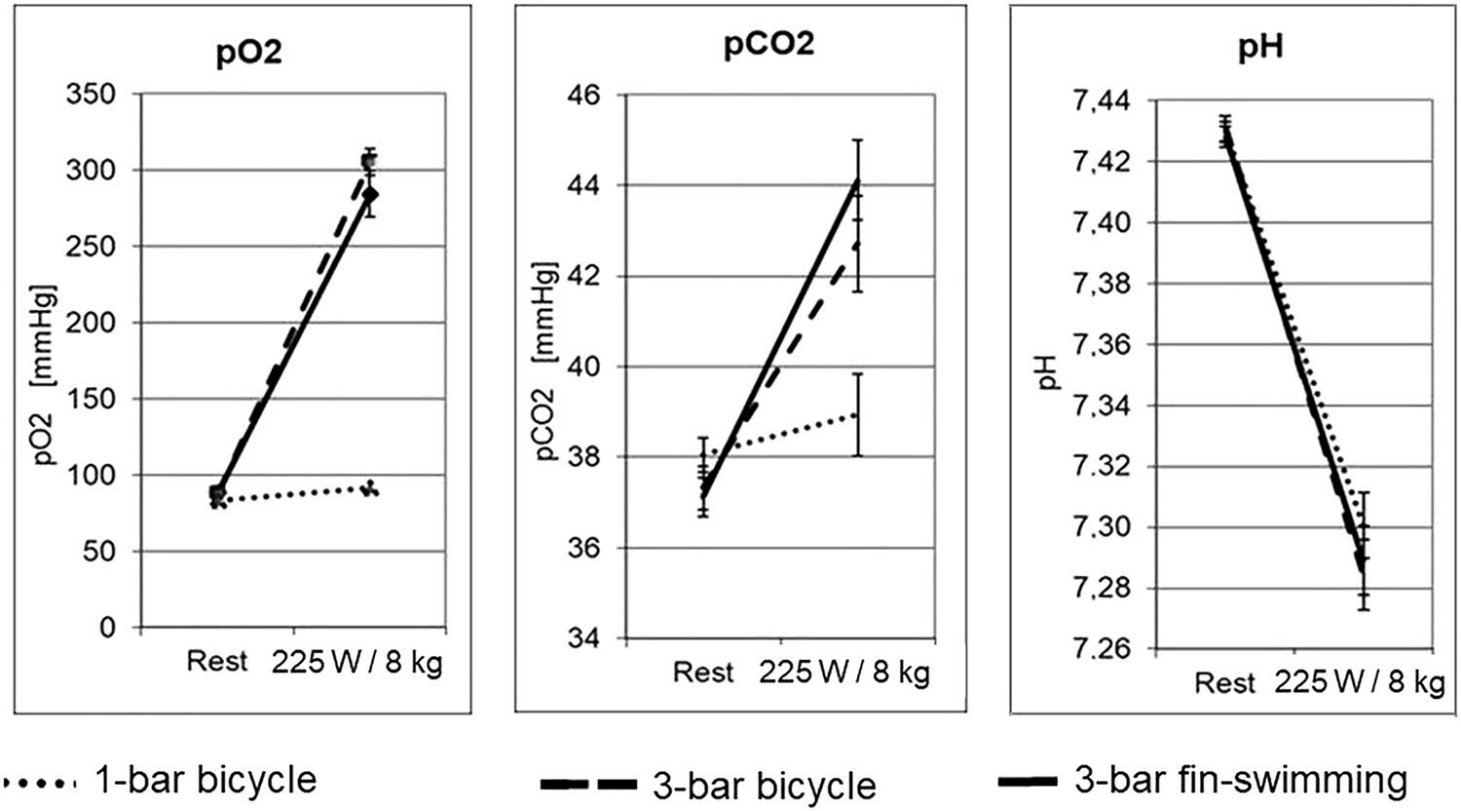
Ergo group: capillary blood gases and pH for bicycle ergometry at 1 bar and 3 bar and for fin-swimming ergometry at 3 bar at rest and at the highest load. Left: *p*O_2_ at rest was normal during all three set-ups. At the highest load and 3 bar ambient pressure, *p*O_2_ had significantly increased, while it was only moderately increased during 1-bar bicycle ergometry that was significantly lower compared with both 3-bar conditions (*p* < 0.05). Middle: *p*CO_2_ at rest was normal with about 37.5 mmHg for all three set-ups. Immediately after the end of 1-bar cycling, *p*CO_2_ was only slightly increased to 38.9 ± 4.5 mmHg. However, *p*CO_2_ after both 3-bar conditions was significantly increased: cycling to 42.7 ± 5.1 mmHg and fin-swimming to 44.1 ± 4.3 mmHg (*p* < 0.05). Right: pH at rest was within the normal range for all three set-ups. At the highest load and both at 1 bar and 3 bar ambient pressure, pH had significantly decreased by almost the same amount (*p* < 0.05). Cave: *y*-axis starts at a value of 7.26

*Blood gases and pH* Blood gases and pH at rest under normobaric conditions were in the normal range under all three set-ups. During 1-bar bicycle ergometry *p*O_2_ in the capillary blood had insignificantly increased to 90 ± 5 mmHg at the highest load. In contrast, at the highest load and for both hyperbaric set-ups, *p*O_2_ had significantly increased due to the increased ambient pressure: 3-bar cycling to 298 ± 11 mmHg and 3-bar fin-swimming to 280 ± 12 mmHg (Fig. 7).

*p*CO_2_ at rest was normal with about 37.5 mmHg for all three set-ups. Immediately after the end of 1-bar cycling, *p*CO_2_ was almost maintained at 38.9 ± 4.5 mmHg. However, *p*CO_2_ after both 3-bar conditions was significantly increased: cycling to 42.7 ± 5.1 mmHg and fin-swimming to 44.1 ± 4.3 mmHg (*p* < 0.05) (Fig. 6). pH at rest was within the normal range for all three set-ups, i.e. around 7.43. At the highest load and both at 1 bar and 3 bar ambient pressure, pH had significantly decreased to a value around 7.29 (*p* < 0.05) (Fig. 6).

*Index of efficiency* The index decreased significantly from the first step (5 kg) to the 6-kg-step for both loads and decreased further with each load step. The difference between the 5-kg-load at 1 bar and 3 bar was significant (7.5 ± 2.3 vs 10.2 ± 2.9) (Fig. 7).

Style group

*Swimming style and index of efficiency* Style of underwater fin-swimming and the index were calculated for each workload step for both the beginners (< 100 dives; *n* = 13; 16–90 dives) and the advanced divers (≥ 100 dives; *n* = 7; 185–831 dives).

**Fig. 8:**
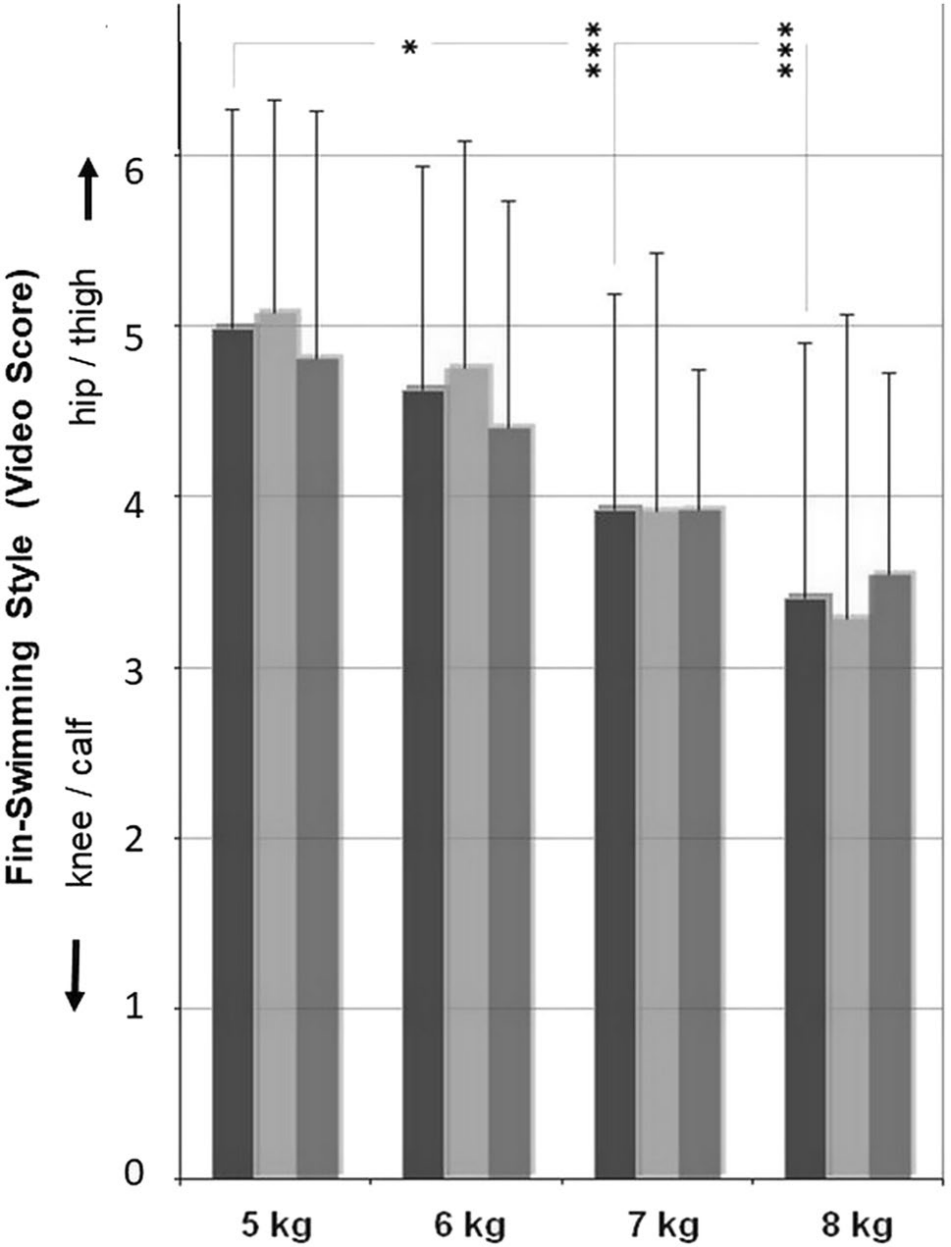
Style group: video-score of fin-swimming style at 3 bar (≙ 20 m depth). With increasing workload highly significant decreases in the video-score towards a more knee/calf-oriented style; for all divers from 5 to 6 kg (*p* < 0.05), from 5 to 7 kg (*p* < 0.001), and from 5 to 8 kg (*p* < 0.001). No differences between beginners and advanced divers. Black columns: all divers; light grey: beginners (< 100 dives); dark grey: advanced divers (≥ 100 dives)

Video-scores of fin-swimming styles changed in all divers significantly from a more hip/thigh-oriented swimming style on the lower workloads (5–6 kg) towards a more knee/calf-oriented style on the high workloads (7–8 kg) without major differences between beginner and advanced divers (*p* < 0.05) (Fig. 8).

**Fig. 9:**
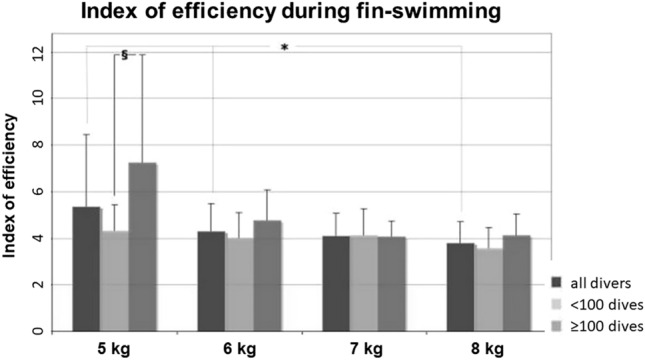
Style group: Index of efficiency of fin-swimming at 3 bar (20 m depth). On the 5 kg load, the index of the advanced divers was significantly better over the beginners (^§^*p* < 0.05). With increasing workload, the index was significantly decreased in all divers (*p < 0.05) with no further differences between beginners and advanced divers

The index of efficiency on each step decreased significantly with increasing weight in all divers from 5.2 ± 3.1 (5 kg) to 3.8 ± 0.9 (8 kg) in parallel with the observed changes in swimming style (*p* < 0.05). However, the index of efficiency of the advanced divers was significantly higher than in the beginners (6.5 ± 3.6 vs. 4.5 ± 1.1) (*p* < 0.05) at the 5 kg workload, but the differences between beginners and advanced divers disappeared with increasing workloads (Fig. 9).

### Ventilation and oxygen uptake

**Fig. 10:**
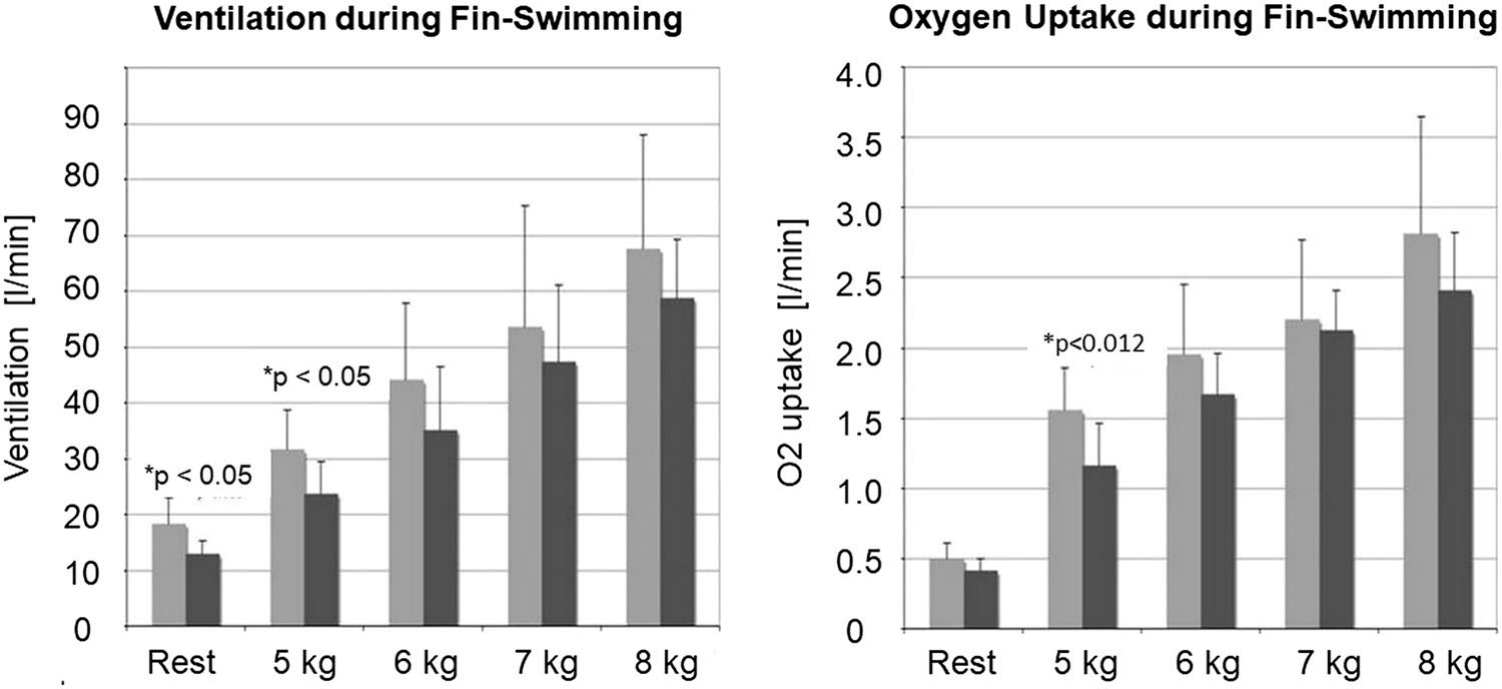
Left: style group: ventilation during fin-swimming at 3 bar (≙ 20 m depth). $\dot{V}$E of the beginners (light grey columns) was higher at rest over advanced divers (dark grey columns) and on all workload steps, differences were significant at rest and on the 5 kg step (*p* < 0.05). Right: oxygen uptake during fin-swimming at 3 bar (≙ 20 m depth). Higher $\dot{V}$O_2_ of the beginners becomes apparent at rest and on all workload steps. Differences were significant on the 5 kg step (*p* < 0.05)

$\dot{V}$E at rest was significantly lower in the advanced divers (13 ± 2.2 l/min vs. 18.3 ± 4.7 l/min; *p* < 0.05) with corresponding but lower differences in $\dot{V}$O_2_. During fin-swimming on the 5 kg-step, both $\dot{V}$E (23.7 ± 5.9 l/min vs 31.5 ± 7.1 l/min; *p* = 0.05) and $\dot{V}$O_2_ (1.2 ± 0.3 l/min vs. 1.6 ± 0.3 l/min; *p* < 0.05), were significantly lower in the advanced divers, indicating CO_2_ retention. The differences became smaller and lost significance with increasing loads (Fig. 10).

### Comparison of calculated bottom times

The individual results of the most experienced (831 dives) and the least experienced (16 dives) diver are displayed in Table 1. Both were well-trained athletes with comparable HR around 120/min on the 6-kg step, and both completed all four workload steps. However, the advanced diver reached a much higher Ix_Eff_ in fin-swimming particularly on the lower workloads by utilising the more efficient hip/thigh-oriented swimming style. Using a 200-bar diving device with double 7-l tanks (≙ 2,800 l) at the depth of 20 m would permit him bottom times of 71.8 min (5 kg) and 58.2 min (6 kg). The unexperienced diver utilised throughout the inefficient knee/calf-oriented swimming style. As a result, his $\dot{V}$E and $\dot{V}$O_2_ were on all steps higher than those of the experienced diver, resulting in a low Ix_Eff_. Hence, the calculated bottom times were substantially shorter with 22.2 min (5 kg) and 13.5 min (6 kg) (Table 1).

**Table 1:** Bottom-times calculated for an air supply of 2800 l and a depth of 20 m

	Rest	6 kg
Best case		
Index of efficiency	–	7.6
$\dot{V}$E [l/min]	10	16
$\dot{V}$O_2_ [l/min]	0.39	1.11
Calculated bottom time [min]	93.3	58.3
Orientation of swimming style	–	Hip/thigh
Worst case		
Index of efficiency	–	2.1
$\dot{V}$E [l/min]	16	69
$\dot{V}$O_2_ [l/min]	0.36	3.23
Calculated bottom time [min]	58.3	13.5
Orientation of swimming style	–	Knee/calf

Index of efficiency and ventilatory parameters are presented for the “best-case” and the “worst-case” in fin-swimming. Best Case: 831 dives. Excellent aerobic training status (HR 119/min at 6 kg). Worst case: 16 dives. Excellent aerobic training status (HR 125/min at 6 kg)

## Discussion

The two main findings of this study are: (1) The suspended-weights system for stationary fin-swimming with variable workloads inside the wet section of the hyperbaric chamber of the HYDRA 2000 allows for simulating free-water diving and fin-swimming under laboratory conditions. To our knowledge, this is the first time that the complex situation of diving with its characteristics of submersion and increased pressures has become available for sports physiological studies permitting the assessment of a great variety of data. (2) A solid diving experience in terms of ventilation and fin-swimming style at rest and during exercise helps preserving the air supply and thus preventing from unexpected running-out-of-air.

### Functional aspects of underwater ergospirometry

The suspended-weights system allows for stationary fin-swimming, and shows similarities to the “tethered-swimming” training, which is an accepted method in elite swimmers´ training for measuring $\dot{V}$O_2_ during swimming in parallel to conventional swim tests (O’Toole and Douglas 1995; Ueda and Kurokawa 1995; Hooper et al. 1998). In both, the tethered swimming- and the suspended-weights ergometry, the effective workload is not defined by the velocity over ground but by the work for counterbalancing the retractive force. This mirrors the effective amount of locomotor force that must be generated by the swimmer or diver.

For the sake of completeness, a shallow water ergometry system is mentioned, where divers can dive along an octagonal parcours at incremental speeds until exhaustion (Steinberg et al. 2011).

Some more aspects are mentioned that can be investigated in the future using the suspended-weights ergometry: greater depths down to 50 m can be visited to assess effects of increased gas densities, fin-types and fin-kicking frequencies can be further optimised (Pendergast et al. 1996). Moreover, in view of professional diving and the advent of technical diving, the effects of gas mixtures like heliox and trimix might deserve studies under laboratory conditions.

### Study limitations

A suspended-weights system in a hyperbaric chamber cannot fully reproduce all aspects of SCUBA diving like normal changes in body-position and the velocity-dependent drag in free motion (Pendergast et al. 2005). In spite of those differences, our results demonstrate that the suspended-weights technique is suitable for investigating the diver’s underwater finning performance at realistic diving depths and with varying workloads. The test situations are reproducible, i.e. changes in the environment as pressure, temperature, visibility can be excluded, and the setting allows for an ergospirometric online-monitoring, since the diver remains stationary during exercise.

The 3-min-step duration in our study differs from approaches focussing on efficiency as these use steps between 5 and 10 min, because they need not only to reach the steady state, but also to keep it for several minutes to calculate a reliable average. We had decided for 3-min steps, because they are commonly used in the area of sports physiology.

Finally, the equivalence between bicycle ergometry and suspended weights is nicely demonstrated via close similarities in heart rate vs load, ventilation, $\dot{V}$O_2_, RER and lactate, *p*O_2_, *p*CO_2_, and pH. Acceptedly, the form drag is lacking while stationary fin-swimming, as well as eddies and turbulences might have had an effect on metabolic energy expenditure.

### Ergo group

This group was supposed to further evaluate properties of the novel underwater fin-swimming ergospirometry system. The results at 20-m simulated depths are in good agreement with earlier results on the thrust force during tethered fin-swimming in shallow water (Yamaguchi et al. 1995), were HR, $\dot{V}$E and $\dot{V}$O_2_ also linearly increased with increasing load.

Unfortunately, the index of efficiency cannot not be translated into values of the work efficiency. However, the work efficiency of cycling in our own group (~ 26%) nicely compared with bicyclists (~ 28%) in an earlier study (Mogensen et al. 2006).

Differences between in-air and underwater bicycle ergometry need mentioning. In one study, the underwater gross capacity was decreased by about 50% compared to a given in-air workload (Almeling et al. 2006), showing that cycling exercise is less efficient underwater than in air (Chen et al. 1996; Vinetti et al. 2021). Likely, such differences are owing to an increased pedal drag and decreased pressure against pedals due to positive buoyancy (Shykoff 2009).

Ergospirometry systems require gradable increases in load for physiological measures, as HR, $\dot{V}$E, and $\dot{V}$O_2_ correlate linearly, at least at moderate loads (Hagerman et al. 1988). At a simulated depth of 20 m, our underwater ergometry fulfilled this criterion, at least in the range from 5 to 8 kg counterbalancing weights. In this range the 1 kg-steps were directly comparable to bicycle workloads from 75 to 225 W, in particular with respect to increases in HR, $\dot{V}$E and $\dot{V}$O_2_. However, this setting has so far not been tested at depths ≥ 40 m. Therefore, the influence of the severely increased breathing work due to the considerably increased density of the breathing gas (Maio and Farhi 1967; Wright et al. 1972) on the linearity needs further studies.

Although the increases in HR and $\dot{V}$O_2_ were statistically not different for all three set-ups, the significant differences in $\dot{V}$E, particularly on the higher workload steps, indicate a major influence of the elevated ambient pressure, i.e. the elevated *p*O_2_, on the breathing characteristics of experienced divers. This influence was independent from dry or underwater conditions. A decrease in $\dot{V}$E during diving or hyperbaric exposure has been described before (Peacher et al. 2010; Tetzlaff et al. 1998), and demonstrates that experienced divers learn to optimise underwater-breathing work particularly during high workloads, as suggested for all organisms (Poon 1987). These divers reduce ventilation under hyperbaric and hyperoxic conditions and develop a tolerance to increased CO_2_-levels resulting from hypoventilation (Kerem et al. 1995; Eynan et al. 2003). The diving beginners of our second group did not exhibit this behaviour. Thus, their $\dot{V}$E was significantly increased already at rest and the lowest workload, while $\dot{V}$O_2_ was increased to a lesser extent.

### Style group

The hip/thigh-oriented style was predominantly used on the lower workload steps and was associated with a better index of efficiency compared to the more knee/calf-oriented style, used on the higher workload steps. We suggest using this latter style increases the drag at each single stroke while the lower leg moves upwards.

No differences were found between beginners and advanced divers with respect to their actual swimming style on the different workloads. However, the advanced divers generally showed compared to expected values both a lower $\dot{V}$E and $\dot{V}$O_2_ already at rest compared to the beginners. The significant differences in $\dot{V}$E, $\dot{V}$O_2_ and index of efficiency on the lower load levels have practical implications, as the intensity of fin-swimming in recreational diving is normally relatively low. Therefore, these differences gain importance for the individual air consumption of recreational divers.

Two mechanisms likely contribute to the differences in $\dot{V}$E and $\dot{V}$O_2_ of beginners and advanced divers. Firstly, the CO_2_-insensitivity (Morrison et al. 1978) or the CO_2_-tolerance (Tetzlaff et al. 1998; Delapille et al. 2001) of advanced divers is increased. In consequence, the elevated CO_2_-levels allow a reduced ventilation during the dive, in particular under hyperbaric/hyperoxic conditions (Pendergast et al. 1996). This would be in good agreement with the advanced divers of our ergo group, where *p*CO_2_-values were significantly elevated particularly while cycling and diving under pressure. Of note: CO_2_ retention, or hypercapnia, is a known risk of diving that can cause mental and physical impairments leading to life-threatening accidents (Dunworth et al. 2017).

With less ventilation, CO_2_-retainment reduces the energy cost of breathing, which is increased during water immersion (Held and Pendergast 2013), and in particular under pressure, because of the elevated gas density. Thus, the relative hypoventilation might contribute to the lower $\dot{V}$O_2_ in experienced divers. It is remembered that there were only experienced divers in the ergo-group, all showing signs of *p*CO_2_-retention under elevated pressure conditions. Moreover, well experienced divers are less subject to underwater hyperventilation, which is often seen in diving beginners and might be due to both the unfamiliar environment and diving gear as well as to changes in breathing mechanics (Muth et al. 2005). As a result, the individual fin-swimming experience will directly affect $\dot{V}$E during exercise. An optimization of the individual movement pattern, e.g. while swimming (Taïar et al. 1999), will be associated with reduced energy costs in both, advanced and beginners.

*Comparison of calculated bottom times* The influence of the fin-swimming technique on underwater air consumption should be highlighted by directly comparing our ‘best case’ and the ‘worst case’: the ‘best case’ was an experienced diver (> 800 dives) with low $\dot{V}$E and $\dot{V}$O_2_ and a hip-thigh oriented swimming style at the 6 kg step. This moderate load compares well with loads of recreational dives. Fin-swimming at a 20-m depth with an air supply of 2800 l resulted in a calculated bottom time of roughly 60 min. The ‘worst case’ diver had much less diving experience (16 dives). His $\dot{V}$E and $\dot{V}$O_2_ were already high at the 6-kg step, and he solely employed the knee-calf-oriented swimming style. Nevertheless, he completed all workloads as his training status was excellent, as well. Fin-swimming at 20 m and also having 2800 l of air, this diver had a greatly reduced calculated bottom time of roughly 14 min, meaning that this diver is at risk to unexpectedly early run-out-of-air at higher workloads.

It is suggested that a combination of an increased CO_2_-tolerance/CO_2_-insensitivity, a reduced tendency for hyperventilation owing to psychologic stress, and a more economic fin-swimming style in the advanced divers warrants a reasonably low $\dot{V}$E. Thus, individual diving experience much affects underwater $\dot{V}$E and thereby the available time for diving activities. On the other hand, other differences might exist between well-trained and untrained diving beginners.

With respect to a sudden running-out-of-air, there might exist an even higher risk for overall well-trained diving-beginners because of their ability to reach very high ventilation values during hard work. They may keep the fin-swimming speed of the group, but empty their tank in unexpectedly short time and thus may run into trouble without preceding warnings.

In conclusion, the results from the first part of the study show that wide-ranging diving-ergospirometry can be realised in a wet diving-chamber and that fin-swimming on depth with suspended weights allows for measuring many sports-physiological variables. In addition, diving ergospirometry in terms of changes in HR, $\dot{V}$E and $\dot{V}$O_2_ in response to increasing loads compare well with traditional bicycle exercise. The second part of the study reveals that both the economy of the fin-swimming style and the breathing characteristics affect the individual air consumption during the dive. Low experience, i.e., an inefficient fin-swimming style and maladapted breathing characteristics are responsible for an unnecessary waste of the most limited underwater resource. Sudden running-out-of-air is one of the most dangerous risks in scuba diving. In turn, due to an increased CO_2_-tolerance/CO_2_-insensitivity hypoventilation is seen in experienced divers. The resulting hypercapnia in CO_2_-retainers must be considered another risk even if it conserves air.

## Abbreviations

HR: Heart rate
Ix_Eff_: Index of efficiency
pCO_2_: Partial CO_2_ pressure
pH: Pondus hydrogenii
pO_2_: Partial O_2_ pressure
RER: Respiratory ratio
SCUBA: Self-contained underwater-breathing apparatus
SD: Standard deviation
$\dot{V}$CO_2_: CO_2_-production
$$\dot{V}$$E: Ventilation
$$\dot{V}$$O_2_: O_2_ uptake
