# Correction to: Diving ergospirometry with suspended weights: breathing- and fin-swimming style matter

**DOI:** 10.1007/s00421-022-05037-8

**Published:** 2022-10-03

**Authors:** Andreas Koch, Dennis Kramkowski, Mattes Holzum, Wataru Kähler, Sebastian Klapa, Bente Rieger, Burkhard Weisser, Jochen D. Schipke

**Affiliations:** 1 German Naval Medical Institute, Section for Maritime Medicine, Kronshagen, Germany; 2 Institute of Experimental Medicine, Section for Maritime Medicine, Christian-Albrechts-University, Kiel, Germany; 3 Dept. of Sportsmedicine, Christian-Albrechts-University, Kiel, Germany; 4 Research Group Experimental Surgery, University Hospital, Düsseldorf, Germany

## Correction to: European Journal of Applied Physiology 10.1007/s00421-022-05009-y

The original version of this article unfortunately contained a mistake. The corrected details are given below for your reading.

The author’s affiliations should be:

Andreas Koch^1,2^ · Dennis Kramkowski^1^ · Matthias Holzum^3^ · Wataru Kähler^1,2^ · Sebastian Klapa^1,2^ · Bente Rieger^1,2^ · Burkhard Weisser^3^ · Jochen D. Schipke^4^

^1^ German Naval Medical Institute, Section for Maritime Medicine, Kronshagen, Germany

^2^ Institute of Experimental Medicine, Section for Maritime Medicine, Christian-Albrechts-University, Kiel, Germany

^3^ Dept. of Sportsmedicine, Christian-Albrechts-University, Kiel, Germany

^4^ Research Group Experimental Surgery, University Hospital, Düsseldorf, Germany

In section “Comparison of calculated bottom times”, second sentence should read as

An air supply of 2800 l (14l at 200bar) was assumed.

The legend for Fig. 2 was inadvertently omitted the following text “© Dr. F. Moeller, DSHS, Cologne”. The figure legend should have appeared as shown below.

Fin swimming styles. Left: hip/thigh style; Right: knee/calf style © Dr. F. Moeller, DSHS, Cologne.

